# Quality assurance in radiotherapy: analysis of the causes of not starting or early radiotherapy withdrawal

**DOI:** 10.1186/s13014-014-0260-0

**Published:** 2014-12-04

**Authors:** Meritxell Arenas, Sebastià Sabater, Marina Gascón, Ivan Henríquez, M José Bueno, Àngels Rius, Àngels Rovirosa, David Gómez, Anna Lafuerza, Albert Biete, Jordi Colomer

**Affiliations:** Department of Radiation Oncology, Hospital Universitari Sant Joan de Reus, Institut d’Investigacions Sanitàries Pere Virgili (IISPV), Universitat Rovira i Virgili (URV), Tarragona, Spain; Department of Radiation Oncology, Complejo Hospitalario Universitario de Albacete, Albacete, Spain; Department of Quality, Hospital Universitari Sant Joan de Reus, Tarragona, Spain; Department of Statistics, Hospital Universitari Sant Joan de Reus, Tarragona, Spain; Department of Radiation Oncology, Hospital Universitari Clínic de Barcelona, Barcelona, Spain; Hospital Universitari Sant Joan de Reus and Group SAGESSA (Assistència Sanitària i Social), Tarragona, Spain

**Keywords:** Quality assurance, Radiotherapy, Not starting treatments, Early withdrawal treatments

## Abstract

**Background:**

The aim of this study was to analyse the reasons for not starting or for early of radiotherapy at the Radiation Oncology Department.

**Methods:**

All radiotherapy treatments from March 2010 to February 2012 were included. Early withdrawals from treatment those that never started recorded. Clinical, demographic and dosimetric variables were also noted.

**Results:**

From a total of 3250 patients treated and reviewed, 121 (4%) did not start or complete the planned treatment. Of those, 63 (52%) did not receive any radiotherapy fraction and 58 (48%) did not complete the course, 74% were male and 26% were female. The mean age was 67 ± 13 years. The most common primary tumour was lung (28%), followed by rectum (16%). The aim of treatment was 62% radical and 38% palliative, 44% of patients had metastases; the most common metastatic site was bone, followed by brain. In 38% of cases (46 patients) radiotherapy was administered concomitantly with chemotherapy (10 cases (22%) were rectal cancers).

The most common reason for not beginning or for early withdrawal of treatment was clinical progression (58/121, 48%). Of those, 43% died (52/121), 35 of them because of the progression of the disease and 17 from other causes. Incomplete treatment regimens were due to toxicity (12/121 (10%), of which 10 patients underwent concomitant chemotherapy for rectal cancer).

**Conclusions:**

The number of patients who did not complete their course of treatment is low, which shows good judgement in indications and patient selection. The most common reason for incomplete treatments was clinical progression. Rectal cancer treated with concomitant chemotherapy was the most frequent reason of the interruption of radiotherapy for toxicity.

## Background

The province of Tarragona in the south of Catalonia, Spain, has a total area of 6303 Km^2^ and a total population over 800000 inhabitants. The Radiation Oncology Department of *Hospital Universitari Sant Joan de Reus* attends to all the candidates for radiotherapy (RT) in the entire province, and is a centre of reference for the Tarragona province.

Quality assurance in health care has become more and more important in recent years. Several papers have been published on quality control in RT [1-6]. Our department has great interest in quality assurance and has put an enormous effort into its quality assurance programme. In 2000, a quality assurance system project based on the so-called *International Organization for Standardizations (ISO) 9001 Quality Standards* was started. One of the standards applied in our quality assurance system was the number of patients with incomplete treatments.

The aim of this study is to analyse the reasons for incomplete treatments at the Radiation Oncology Department.

## Methods

All treatments carried out at the Radiation Oncology Department over a two-year period (from March 2010 until February 2012) were reviewed. Early withdrawals and those patients that never started treatment were recorded. Information regarding patients, demographic characteristics, disease, treatment characteristics and dosimetric variables were collected retrospectively from the patients' records.

Our department receives patients for RT treatments from different specialists from seven hospitals in the province. We have a hospitalisation area where the patients' who need care are attended. Patients are admitted for symptom control, for disease progression or for toxicity treatment.

The planning process for RT includes the first consultation including collecting the clinical history and examination of the patient. In this first consultation the clinician explains the aim of treatment and its procedures and informs patients and their families of the benefits and any acute and/or late side effects they might expect and patients sign the treatment consent form. That same day, if the indicated treatment proceeds, the clinical decides the position and immobilization of the patient simulation and the acquisition of CT simulation. Then we proceed to the prescription dose treatment and delineation of tumour volumes and organs at risk, in order to make the corresponding 3D dosimetry. Then we review dosimetry, the treatment volumes as well as histograms of the organs at risk. If treatment is accepted, the patient starts treatment in the unit prior to performing verification.

The RT indication, radical vs. palliative, as well as the need of a concomitant chemotherapy course is evaluated by the attending clinician after assessment of general conditions and co-morbidities of the patients. During treatment, patients are scheduled for regular visits.

In our review, patients had been treated by standard techniques at normo- or hypofractionation (1.8-8 Gy/day) 5 days/week with a variety of equipment including 6–18 MV photons and/or 6–20 MeV electrons from the linear accelerator. Our Department has 4 linear accelerators, 1 ortho-voltage, and a high-dose brachytherapy programme. All treatment planning was done in 3D.

In this study, we define incomplete treatment as a patient who has undergone at least one simulation/planning procedure prior to starting an RT course; early withdrawal means that a patient has received some irradiation treatment fraction but failed to complete the scheduled course; a non-starting patient is defined as one who did not receive even a single fraction of radiation after the simulation and planning. A compliant patient is therefore defined as a patient who was able to complete the course of radiation as intended.

### Statistical analysis

The results are shown as medians (± standard deviations). Chi-square test was used to compare categorical variables. Two-sided t-test was used to determine differences in continuous variables. In the logistic regression model, we included all predictor variables in the univariate analysis. Values of p <0.05 were considered significant.

## Results

A total of 3250 patients were treated during the analysed period, RT was indicated (most frequent first) to treat breast cancer (n = 748 patients, 23%), to improve symptoms (n = 715, 22%), to treat prostate cancer (n = 585, 18%), and to treat rectal cancer (n = 221, 7%).

Table 1 shows patient characteristics of all incomplete treatments. It shows that 121/3250 patients (4%) were incomplete treatments. Of those, 63/121 (52%) did not receive any therapy fraction and the other 58/121 (48%) did not complete the scheduled course, 74% of them were male and 26% were female. The mean age was 67 ± 13 years. Mean distance from home to Department was 11 ± 16 Km. Fifty-eight percent of the patients travelled to the Department by ambulance and 27% of them were in-patients. The most common primary tumour location was the lung (28%), followed by the rectum (16%). The aim of treatment was radical in 62% and palliative in 38% of cases. Metastases were evident in 44% of the patients. The most common metastatic site was bone, followed by brain. In 38% of cases (46/121 patients) RT was administered concomitantly with chemotherapy (10/121 cases (22%) were rectal cancers).

**Table 1:** **Patient characteristics of all incomplete treatments**

		**N**	**%**
**Sex**	Male	89	74
	Female	32	26
**Tumour location**	Lung	34	28
	Rectum	19	16
	Skin	14	12
	Other	54	45
**Metastasis**	Yes	41	34
	No	80	66
**Palliative**	Yes	46	38
	No	75	62
**Concomitant chemotherapy**	Yes	46	38
	No	75	62
**Ambulance**	Yes	70	58
	No	51	42
**Inpatient**	Yes	33	27
	No	88	73
**Type of early withdrawal**	Incomplete course	58	48
	Any fraction	63	52
**Reason for withdrawal**	Death due to tumour progression	35	29
	Clinical progression	23	19
	Death due to non-oncology reasons	17	14
	Toxicity	12	10
	Patient refusal	10	8
	Dosimetric issues	5	4
	Other	19	16

Mean time to the beginning of treatment was 7 ± 4 days. Mean planned dose was 47 ± 18 Gy. Mean sessions scheduled 22 ± 12 Gy. Early withdrawal median dose 12 ± 17 Gy. Clinical progression was the most frequent reason for incomplete courses (58/121, 48%). Patient death was the cause of incomplete courses in 52 patients (43%), 35 of them due to clinical progression and 17 from other causes. Toxicity was responsible for incompleteness in 10% (12/121) of patients, ten of them being rectal cancer patients treated with concomitant chemotherapy.

Table 2 compares the characteristics between to non-starting patients and early withdrawal patients.

**Table 2:** **Characteristics of non-starting patients and early withdrawn patients**

		**Non-starting**		**Early withdrawal**		
		N	%	N	%	
**Sex**	Male	50	79	39	67	0.131
	Female	13	21	19	33	
**Tumour location**	Lung	21	33	13	22	0.045
	Rectum	4	6	15	26	
	Skin	5	8	9	16	
	Other	33	52	21	36	
**Metastasis**	Yes	16	25	25	43	0.04
	No	47	75	33	57	
**Palliative**	Yes	19	30	27	47	0.063
	No	44	70	31	53	
**Concomitant chemotherapy**	Yes	21	33	25	43	0.269
	No	42	67	33	57	
**Ambulance**	Yes	30	48	40	69	0.022
	No	32	52	18	31	
**Inpatient**	Yes	12	19	21	36	0.034
	No	51	81	37	64	
**Reason for withdrawal**	Death due to tumour progression	17	27	18	31	
	Clinical progression	8	13	15	26	
	Non-oncology reasons	6	10	11	19	0.001
	Toxicity	2	3	8	14	
	Patient refusal	10	16	2	3	
	Dosimetric issues	5	8	0	0	
	Other	15	24	4	7	

We compared the non-compliant irradiation group with the patients that finished their planned RT course. Men were more frequently associated with non-compliant irradiation courses (5%) than women (2%) (p < 0.005). A slightly advanced age was significantly associated with non-compliance (non-compliant courses, 67 ± 13 years, completed courses, 65 ± 13 years). Palliative patients were significantly more non-compliant than non-palliative patients (6% vs. 3%; p < 0.005) but palliative patients were slightly significantly younger (64 ± 13 years) than radical patients (65 ± 13 years) (p = 0.049). There was a significant association between the primary tumour location and non-compliance; the main locations were brain (10% of them), lung (8% of them), rectum (7% of them) and head and neck (6%) (p < 0.005). Despite that, a palliative course was associated with bladder (59% of them), lung (58% of them), rectum (17% of them), and breast (15% of them) (p < 0.005).

No significant differences in the planned dose were evident between patients who completed their irradiation course (48 ± 21 Gy) and those non-compliant (47 ± 18 Gy). Mean dose in the non-compliant course group, 12 ± 17 Gy. No significant differences between non-starting patients and early withdrawal patients were evident according to the age, sex, location of the primary tumour, or planned dose.

Table 3 summarizes the results of the logistic regression analysis for predictors of non-compliance among all patients. Age, ambulance usage and primary tumour location were significant. Sex and type of RT course were not predictive of treatment discontinuation in the multivariate analysis.

**Table 3:** **Multivariate logistic regression for predictors of lack of treatment compliance among all patients**

	**B**	**Exp (B)**	**95% CI**	**Sig.**
RT course (radical)	.354	1.424	.913 – 2.222	.119
Age	-.021	.980	.964 - .996	.013
Ambulance use	-.982	.374	.256 - .547	.000
Sex (female)	.245	1.278	.783 – 2.085	.326
Primary location				.000
Bladder	1.499	4.478	1.285 – 15.605	.019
Head & neck	.345	1.412	.655 – 3.044	.378
Skin	1.837	6.276	2.475 – 15.913	.000
Breast	1.992	7.329	2.977 – 18.046	.000
Lung	.252	1.287	.713 – 2.323	.403
Rectum	.289	1.335	.662 – 2.693	.420
Lymphoma	1.190	3.287	.732 - 14.751	.120
Prostate	2.256	9.546	3.834 – 23.764	.000
Gynaecological	18.517	110098206.961	.000 - .	.995
CNS	-.165	.847	.329 – 2.185	.732

RT: radiotherapy; CNS: Central nervous system; 95% CI: 95% confidence interval.

## Discussion

Radiation treatment is a complex procedure because it requires highly specialized equipment and technology as well as trained professionals.

Both the patient-related aspects (diagnosis, selection, treatment indication, justification, referral, planning, therapy, and follow-up) and the control and measurement procedures that form the technical part of the treatment process should be subject to regular planning, verification and, mostly, constant improvement [7,8].

In this study, we focus on incompleteness of radiation courses due to the well-established connection between cancer treatment compliance and its outcomes [9-11]. A correct evaluation of the parameters involved in non-compliance has consequences, not only when selecting patients for a radical/palliative treatment, but also when planning RT resources and saving costs. The causes of non-compliance to a prescribed treatment could be a good quality indicator for an RT department because they can show the accuracy of selection when prescribing irradiation.

Interruptions during the course of treatment include planned unit maintenance and servicing, acute patient toxicity and unexpected malfunction of linear accelerators. Toxicity increases alongside concomitant chemo-radiotherapy schedules. As far as we know, there are no similar global studies published that have analysed early withdrawal and non-starting irradiation factors in the RT domain. Studies have focused on oncological non-compliance treatments, analysing chemotherapy or hormonal therapy or RT in a specific context. Some of the most common topics are breast cancer [12-16] or head and neck cancer [17,18] and insurance status [13,14,17-20], which is related with the hospital type or about elderly patients [16,18,21]. A global study directed at analysing the duration of curative RT has also been published [22], but it was aimed at evaluating ways to counteract short RT interruptions, such as those caused by public holidays or machine maintenance. Some factors associated with treatment interruptions or early withdrawal, described in these published studies, especially those related with health insurance or financial issues, could not be included in our study due to the big differences between the Spanish and American Health Systems and the population sizes.

No irradiation compliance, as defined in the present study, was very low (4%), the main causes being clinical progression (48%) and death due to non-oncology reasons. Male patients were more frequently non-compliant in palliative treatments. Despite results showing a significant slightly younger age for non-compliant patients, the difference has no clinical relevance but is explained by a the younger age of palliative patients. This is an important finding because some studies have associated advanced age with lower frequency of definitive or adequate cancer treatment in some studies [23,24] even though other studies suggest that older patients can benefit similarly to younger patients [25,26] with high rates of irradiation completion [16,18,21]. It is important to keep one’s mind on carrying out an accurate comprehensive geriatric assessment because there is a possibility of declining functional status [27] or even an early death [28] due to cancer treatment in older patients.

Not surprisingly, patients with brain cancer or lung cancer were more frequently non-compliant. A previous report from our group showed a 27% early withdrawal from RT among brain metastasis patients [29] stressing the need for an accurate patient selection in order to avoid over-treatment. Obviously the current study, which involves curative and palliative patients, shows lower figures, with a 4% overall incidence of non-compliance. It should be noted that concomitant chemotherapy for rectal cancer was the most frequent reason for non-compliance due to toxicity, nearly half of them by concomitant capecitabine, despite different studies showing that the replacement of 5-FU by oral capecitabine in concurrent treatment of locally advanced rectal cancer with RT is generally well-tolerated and effective [30-34]. Interestingly, we found that the mean time for beginning treatment was 7 ± 4 days, so the results presented here do not reflect delays due to the health care system or waiting lists.

The main limitation of our study is the lack of additional socio-economic or clinical data that might be useful for designing preventive policies that avoid irradiation discontinuation. Policies directed at predicting, correcting and preventing treatment interruptions, including integrated oncology care pathways, would reduce treatment variations and improve quality of patient care and compliance with guidelines [35]. Central review systems that have demonstrated an increase in CTV delineation [36] could be expanded to clinical areas. In spite of finding a slightly younger age associated with non-compliance, there is a need for a comprehensive geriatric assessment because it has shown to improve elderly cancer patient care. A successful implementation of such measures would result in improved patient outcome as well as a more economical usage of resources.

## Conclusions

This is the first study to our knowledge that reports the incidence and the reasons for incomplete irradiation courses. The overall number of non-compliant of patients is low, showing good judgement in treatment and patient selection.

The most frequent reason for incomplete treatment was clinical progression. Toxicity during a rectal cancer treatment, with concomitant chemotherapy, was the most frequent reason for the interruption of curative courses.

### Summary

The reasons for not starting or early withdrawal of radiotherapy are normally clinical and are not well established. We analysed the patients who did not start radiotherapy or who withdrew early at the Radiation Oncology Department of University Hospital over a two year period. We observed that the most common reason was clinical progression. Rectal cancer treated with concomitant chemotherapy was the most frequent reason for the radiotherapy to be interrupted due to toxicity.
